# Frameshift mutation hotspot identified in Smith-Magenis syndrome: case report and review of literature

**DOI:** 10.1186/1471-2350-11-142

**Published:** 2010-10-08

**Authors:** Hoa T Truong, Tracy Dudding, Christopher L Blanchard, Sarah H Elsea

**Affiliations:** 1 School of Biomedical Sciences, Charles Sturt University, Wagga Wagga, NSW, Australia; 2 Hunter Genetics, Warratah, NSW, Australia; 3 University of Newcastle, Newcastle, NSW, Australia; 4 Departments of Pediatrics and Human Genetics, Virginia Commonwealth University, Richmond, VA, USA; 5 Current Address: KeyGene N.V., Wageningen, The Netherlands

## Abstract

Smith-Magenis syndrome (SMS) is a complex syndrome involving intellectual disabilities, sleep disturbance, behavioural problems, and a variety of craniofacial, skeletal, and visceral anomalies. While the majority of SMS cases harbor an ~3.5 Mb common deletion on 17p11.2 that encompasses the retinoic acid induced-1 (*RAI1*) gene, some patients carry small intragenic deletions or point mutations in *RAI1*. We present data on two cases of Smith-Magenis syndrome with mutation of *RAI1*. Both cases are phenotypically consistent with SMS and *RAI1 *mutation but also have other anomalies not previously reported in SMS, including spontaneous pneumothoraces. These cases also illustrate variability in the SMS phenotype not previously shown for *RAI1 *mutation cases, including hearing loss, absence of self-abusive behaviours, and mild global delays. Sequencing of *RAI1 *revealed mutation of the same heptameric C-tract (CCCCCCC) in exon 3 in both cases (c.3103delC one case and and c.3103insC in the other), resulting in frameshift mutations. Of the seven reported frameshift mutations occurring in poly C-tracts in *RAI1*, four cases (~57%) occur at this heptameric C-tract. Collectively, these results indicate that this heptameric C-tract is a preferential hotspot for single nucleotide insertion/deletions (SNindels) and therefore, should be considered a primary target for analysis in patients suspected for mutations in *RAI1*. We expect that as more patients are sequenced for mutations in *RAI1*, the incidence of frameshift mutations in this hotspot will become more evident.

## Background

Smith-Magenis syndrome (SMS, OMIM182290) is a complex syndrome involving intellectual disabilities, sleep disturbance, behavioural problems, and a variety of craniofacial, skeletal, and visceral anomalies (Table 1). The majority of SMS cases harbor an ~3.5 Mb common deletion on 17p11.2 that encompasses the retinoic acid induced-1 (*RAI1*) gene, which is the causative gene in this disorder [1,2]. Patients who do not have a cytogenetically detectable deletion of 17p11.2 but whom exhibit phenotypes consistent with SMS are sequenced for mutations in *RAI1*. To date, fourteen patients with point mutations or small deletions within *RAI1 *have been reported in the literature (Table 2) [1,3-6].

**Table 1:** Phenotypic features of Smith-Magenis syndrome patients with a 17p11.2 deletion or *RAI1 *mutation.

Clinical findings	17p11.2 deletion (%)*	*RAI1 *mutation (%)*	SMS324	SMS335
**Craniofacial/skeletal**				
Brachycephaly	89	81.8	+	+
Midface hypoplasia	92	72.7	+/-	+
Prognathism (relative to age)	53	88.8	-	-
Tented upper lip	73	91.6	+	-
Broad square face	81	90.9	+	+
Synophyrys	62	33.3	+	-
Cleft lip/palate	9	0	-	-
Brachydactyly (short fingers, toes)	85	83.3	+	+
Short stature	69	9	+	+
Scoliosis	49-67	36.3	+	+
**Otolaryngologic**				
Chronic ear infections	85	54.5	N	+
Hearing loss	68	10	+	+
Horse, deep voice	80	100	+	-
**Neurological/behavioral**				
Intellectual disability	100	100	+	+
Speech delay	>90	70	+	+
Motor delay	>90	70	-	+
Hypotonia	>90	61	+	+
Seizures by history	11-30	16.6	+	-
Sleep disturbance	70-100	100	+	+
Self-hugging/hand-wringing	70-100	100	-	-
Attention-seeking	80-100	100	+	+
Self-injurious behaviours	78-96	100	+	+
Onychotillomania	25-85	80	-	+
Polyemboilokomania	25-85	90	-	+
Head-banging/face-slapping	71	60	+	-
**Ocular**				
Myopia	53	60	+	-
Strabismus	50	40	+	+

*Modified from Girirajan et al. 2007; **+ **= present, **- **= absent, **N **= unknown/not evaluated

**Table 2:** Mutations identified in exon 3 of *RAI1*.

	Nucleotide change	Amino acid change	Mutation
**Mutations reported in Exon 3 of *RAI1***	c.253del19^a^	p.Leu85fsX60	Frameshift
	c.1449delC^b, †^	p.Pro483fsX34	Frameshift
	c.2773del29^b^	p.Val925fsX8	Frameshift
	c.3103insC^c, d, †^	p.Gln1035fsX30	Frameshift
	c.3103delC^e, f, †^	p.Gln1035fsX28	Frameshift
	c.3801delC^a, †^	p.Pro1267fsX46	Frameshift
	c.5265delC^b, †^	p.Pro1755fsX74	Frameshift
	c.1119delC^g, †^	p.Ser373fsX65	Frameshift
	c.4649delC^g, †^	p.Ser1550fsX36	Frameshift
	c.4933delGCCG^g^	p.Ala1645fsX35	Frameshift
	c.2878C>T^c^	p.Arg960X	Nonsense
	c.3634A>G^e^	p.Ser1212Gly	Missense
	c.4685A>G^a^	p.Gln1562Arg	Missense
	c.5423G>A^a^	p.Ser1808Asn	Missense

^a^Girirajan *et al*. 2005, ^b^Slager *et al*. 2003, ^c^Bi *et al*. 2004, ^d^SMS334, ^e^Bi *et al*. 2006, ^f^SMS324, ^g ^Girirajan et al., 2006, ^†^Frameshift mutations resulting from SNindel in poly C-tracts.

## Case Presentation

### Case 1

**Figure 1:**
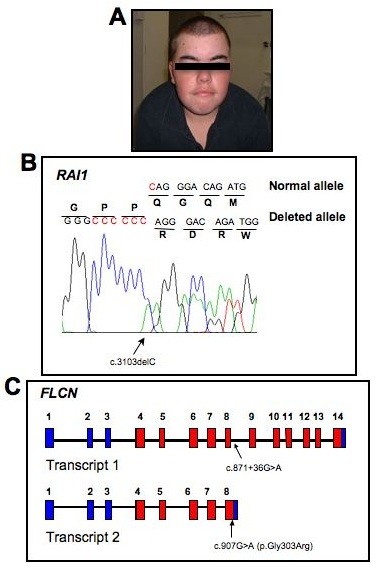
**Mutation screening of SMS324 for RAI1 and FLCN. A.  **Photograph of SMS324 at 18y, exhibiting features characteristic of SMS.  **B.**  DNA chromatograph of the normal allele and the c.3103delC change identified, resulting in a frameshift in the mutant allele.  This mutation is located within a heptameric C-tract (red C’s in the normal allele).  **C. ** Representation of the gene structure of FLCN transcript variant 1(top) and transcript variant 2 (bottom).  Non-coding exons are blue while coding exons are red.  The FLCN polymorphism identified in this patient is localized with arrows in both transcript variants and is numbered accordingly.

SMS324 is an 18-year-old male who was delivered at 38 weeks gestation following premature labor and antepartum haemorrhage. His birth weight was 2,750 g (10^th^-50^th ^centile). While his neonatal period was complicated by gastroesophageal reflux and failure to thrive, he was admitted to the hospital at 1 year of age for being considerably overweight, with fat folds on his arms and legs. He spoke his first words at 6 months and walked at 12 months. His medical history includes two episodes of severe asthma, petit mal seizures between the ages of 5-10 y and three spontaneous pneumothoraces (SP). He had a square shaped face, upslanting palpebral fissures, down turned mouth, inverted upper lip, and synophrys (Fig. 1A). Other physical anomalies included brachydactyly, bilateral fifth finger clinodactyly with a small middle phalanx of his fifth fingers, and pes planus. At 16 years of age, height was 163 cm (< 3^rd ^centile), head circumference was 54 cm, and he presented with relative truncal obesity.

Behaviorally, SMS324 exhibited head-banging and rage attacks starting at age 3. At age 16, he continues to have recurrent episodes of rage attacks, anxiety, and obsessional behavior. He has never slept through the night, with recurrent 3 a.m. wakenings. Apart from finger chewing, he exhibits no self-injurious behaviors, self hugging, onychotillomania or polyembolokomania, which are common behaviors found in SMS patients. He has had a persistent history of sleep disturbance. A formal developmental assessment indicated a mild global developmental delay. He was initially diagnosed with attention deficit disorder; however, a later assessment indicated this diagnosis was incorrect. He was initially mainstreamed in a regular classroom at school but at age 16 now functions in an OI classroom with supervision. The patient was referred for SMS genetic evaluation based upon phenotypic similarities to SMS and/or *RAI1 *mutation patients (Table 1).

Karyotype analysis and 17p11.2 FISH including *RAI1 *for this patient was normal, as were 22q11.2 FISH and subtelomeric deletion screening. DNA sequencing of *RAI1*, however, revealed a deletion of a single cytosine, c.3103delC, in a heptameric C-tract (CCCCCCC) of exon 3 (Fig. 1B). This frameshift mutation predicts the misincorporation of 28 amino acids before a premature stop codon is introduced. The resulting truncated protein is predicted to be non-functional, contributing to the haploinsufficiency of *RAI1*.

### Case 2

SMS335, a 16-year-old female, was delivered full term with a birth weight of 3,398 g (90^th ^percentile). The pregnancy was unremarkable aside from maternal Macrodantin use for urinary tract infection throughout. At the time of evaluation, SMS335 was short in stature (10^th ^-25^th ^percentile), noted to have generalized hypotonia, global developmental delays, significant hearing loss, truncal obesity, and commonly observed behaviours of SMS including sleep disturbance, skin picking, and toenail removal. For SMS335, phenotypes consistent with SMS/mutations in *RAI1 *are noted in Table 1. Chromosome analyses for fragile-X syndrome, Prader-Willi syndrome and SMS were normal for this patient. DNA sequencing performed by GeneDx (Gaithersburg, MD) identified a c.3103insC, located within the same heptameric C-tract in exon 3 of *RAI1*, as noted in SMS324.

## Conclusions

Single nucleotide insertion/deletions (SNindels) occur at an estimated frequency of 1 for every 11,274 bp of the human genome, with more than half occurring in regions with mononucleotide repeats. SNindels can arise from slipped mispairing in regions with repeating units during DNA synthesis or repair [7]. The c.3103delC and c.3103insC frameshift mutations reported here were previously reported as novel mutations in two other patients [4,5]. Of the seven reported frameshift mutations occurring in poly C-tracts in *RAI1*, four cases (~57%) occur at this heptameric C-tract (Table 2). Collectively, these results indicate that this heptameric C-tract is a preferential hotspot for SNindels resulting in a frameshift mutation, and therefore, should be considered a primary target for analysis in patients suspected for mutations in *RAI1*. We expect that as more patients are sequenced for mutations in *RAI1*, the incidence of frameshift mutations in this hotspot will become more evident (*RAI1 *sequencing conditions are available upon request).

Case SMS324 has provided us with an opportunity to evaluate a possible genetic link underlying his history of spontaneous pneumothoraces (SP), a phenotype that has not been documented in any individual with SMS or mutations in *RAI1 *evaluated to date. Mutations in the folliculin (*FLCN*) gene have been implicated in Birt-Hogg-Dubé syndrome (BHD, OMIM135150), an autosomal dominant genodermatosis which predisposes for multiple fibrofolliculomas, renal carcinoma, lung cysts and SP [8-11]. Although SMS324 does not have any other features consistent with BHD aside from the three episodes of SP, clinical heterogeneity is known to exist amongst individuals with germline mutations in the *FLCN *gene. Cases have been reported whereby patients with mutations of *FLCN *exhibit SP without the involvement of fibrofolliculomas or renal tumours [9,12-14].

*FLCN *is located within the SMS region of chromosome 17 and most cases of SMS due to 17p11.2 deletion are heterozygously deleted for this gene. It is alternatively spliced and results in two transcript variants that encode different isoforms. *FLCN *transcript variant 1 (RefSeq NM_144997) encodes the longer isoform of this protein and is widely expressed in various tissues. Transcript variant 2 (NM_144606) uses an alternative splice site in the 3' coding region to produce a shorter protein product. Direct sequencing of the entire coding region of *FLCN*, using primers designed based upon the RefSeq, was performed for SMS324. No known causative mutations were identified. However, we detected a single nucleotide alteration of unknown significance within an intronic sequence in the primary transcript, c.871+36G>A, which is considered to be a non-synonymous variant in *FLCN *(Fig. 1C; dbSNP: rs3744124) [Gunji et al., 2007; Cho et al., 2008; Wei et al., 2009]. Although the coding region of *FLCN *is highly conserved across vertebrate species, rs3744124 mapped to a region of low conservation amongst 17 vertebral species [Cho et al., 2008].

Alignment of the *FLCN *polymorphism found in SMS324 against the sequence of transcript variants 1 and 2 reveals a noteworthy observation. This polymorphic change occurs within intron 8 of transcript variant 1 and is expected to have no effect on the production of a fully functional FLCN isoform 1 protein in SMS324. However, when aligned against transcript variant 2 a nucleotide change, c.907G>A, occurs in coding exon 8 for variant 2, resulting in a missense mutation, p.Gly303Arg (Fig. 1C). Thus, while this DNA alteration is considered an intronic SNP for variant 1, it creates a missense mutation in variant 2. Whether or not this discrepancy is significant or has any bearing on the SP observed in SMS324 is unclear at this time. Interestingly, no studies have investigated the frequency and/or expression of transcript variant 2 in normal or neoplastic tissues and moreover, if there is an isoform specific association with certain phenotypes manifested in BHD, such as SP.

These studies add two interesting cases to the growing list of mutations in *RAI1 *that result in SMS and highlight that key phenotypic differences between patients may exist, even when the underlying etiology is the same. Data presented here highlight a specific poly C tract region within *RAI1 *that may be more prone to single basepair insertion or deletion, with 57% of C tract *RAI1 *mutations reported to date. The cases presented also show that even though *RAI1 *is the primary gene causative of features of SMS, that other genes must be considered in phenotypically complex cases to provide full recurrence and disease risk information for other members of the family.

## Competing interests

The authors declare that they have no competing interests.

## Authors' contributions

All authors contributed to the work presented and participated in writing or reviewing the manuscript. HTT performed the experiments and drafted the initial manuscript; TD identified cases and collected clinical data; CLB analysed molecular data and co-supervised the project; and SHE co-supervised the project, analyzed data, and drafted the final manuscript.

## Consent

Written informed consent was obtained from the patient for publication of this case report and any accompanying images. A copy of the written consent is available for review by the Editor-in-Chief of this journal.

## Pre-publication history

The pre-publication history for this paper can be accessed here:

http://www.biomedcentral.com/1471-2350/11/142/prepub
